# Ants (Hymenoptera: Formicidae) in Vineyards That Are Infested or Uninfested With *Eurhizococcus brasiliensis* (Hemiptera: Margarodidae) in Southeastern Brazil

**DOI:** 10.1093/jisesa/ieu004

**Published:** 2014-01-01

**Authors:** Catarina De Bortoli Munhae, Maria Santina De Castro Morini, Odair Correa Bueno

**Affiliations:** ^1^ Centro de Estudos de Insetos Sociais, Instituto de Biociências, São Paulo State University (UNESP), Rio Claro, São Paulo, Brazil; ^3^ Núcleo de Ciências Ambientais, Laboratório de Mirmecologia, Universidade de Mogi das Cruzes, Mogi das Cruzes, São Paulo, Brazil

**Keywords:** mealybug, ant community, *Solenopsis invicta*, viticulture, subterranean trap

## Abstract

The association between ants and mealybugs can result in damage to agriculture, including vineyards. In southern Brazil, the ant *Linepithema micans* F. contributes to the dispersal of *Eurhizococcus brasiliensis* (Wille) (ground pearl), a root mealybug that can lead to economic losses. In this study, the ant communities in vineyards that were infested or uninfested with ground pearls were evaluated in the primary municipalities that produce the Niágara Rosada variety of grapes in southeastern Brazil. The hypothesis of this study was that the composition of the ant community differs between vineyards with and without *E. brasiliensis.* The ants were collected using subterranean traps in 10 vineyards infested with this mealybug and 10 uninfested vineyards. There was no significant association between ground pearls and the composition or richness of the ant species. *Solenopsis invicta* (Buren) (Hymenoptera: Formicidae) was the most frequently observed, and *Pheidole aberrans* (Mayr), *Pheidole subarmata* (Mayr) *,* and *Brachymyrmex incisus* F. were common, especially in the rainy season when ground-pearl nymphs were prevalent in the state of São Paulo. Species from preserved or specialized environments were recorded in the vineyards, even with the use of conventional management techniques.

## RESUMEN

A associação entre formigas e cochonilhas pode ocasionar prejuízos à agricultura, incluindo parreirais. Na região sul do Brasil, a formiga *Linepithema micans* promove a dispersão de *Eurhizococcus brasiliensis* (pérola-da-terra), uma cochonilha de raiz que pode ocasionar perdas econômicas. Neste estudo, avaliaram-se as comunidades de formigas em parreirais infestados e não infestados pela pérola-da-terra nos principais municípios produtores de uva da variedade Niágara Rosada da região sudeste do Brasil, tendo como hipótese que a composição da comunidade de formigas difere entre os parreirais com e sem *E. brasiliensis* . As formigas foram coletadas usando armadilhas subterrâneas, em dez parreirais infestados e dez não infestados pela cochonilha. Não houve associação significativa entre a pérola-da-terra e a riqueza e composição de espécies de formigas. *Solenopsis invicta* (Hymenoptera: Formicidae) foi a espécie mais frequente e *Pheidole aberrans* , *Pheidole subarmata* e *Brachymyrmex incisus* foram frequentes, principalmente na estação chuvosa, quando as ninfas da pérola-da-terra prevalecem no Estado de São Paulo. Espécies de ambientes mais conservados ou especialistas foram registradas na vitivinicultura, mesmo com o uso de técnicas de manejo convencional.

Viticulture in Brazil includes the south, southeast, central-west, and northeast regions. The state of São Paulo is the largest national producer of table grapes, especially the Niágara Rosada variety ( Protas and Camargo 2011 ).

One of the major viticulture pests in the southern region of Brazil is the root mealybug, *Eurhizococcus brasiliensis* (Wille) (Hemiptera: Margarodidae), referred to as the ground pearl ( Hickel et al. 2008 ), and in the state of São Paulo, it was first recorded in the 1980s ( Lourenção et al. 1989 ). The primary means of dispersal of this insect between vineyards can occur at the first nymphal stage by means of agricultural equipment and rooted plants that were previously contaminated with mealybugs ( Botton et al. 2008 ).

Cultivars with mealybugs contribute to the establishment of ant nests, which maintain a trophobiotic relationship with these Hemiptera due to the release of honeydew ( Chong et al. 2011 ). The galleries built by ants in the soil contribute to the survival of ground pearls in grape cultivations and their subsequent spread ( Botton et al. 2005 ). *Linepithema micans* F. disperse the newly hatched mealybug nymphs in the state of Rio Grande do Sul ( Nondillo et al. 2010 , Martins et al. 2012a ), and two haplotypes are strongly associated with the ground pearl ( Martins et al. 2012a ).

This study investigated the ant communities in vineyards infested or uninfested with *E. brasiliensis* during different seasons. The hypothesis was that the composition of the ant communities differs between vineyards with and without this mealybug. It was expected that cultivars infested with this insect will have one or more species of ants present at high frequencies and because many species of ants farm Hemiptera to feed on their sugary honeydew.

## Materials and Methods

### Collection Areas

**Fig. 1.**
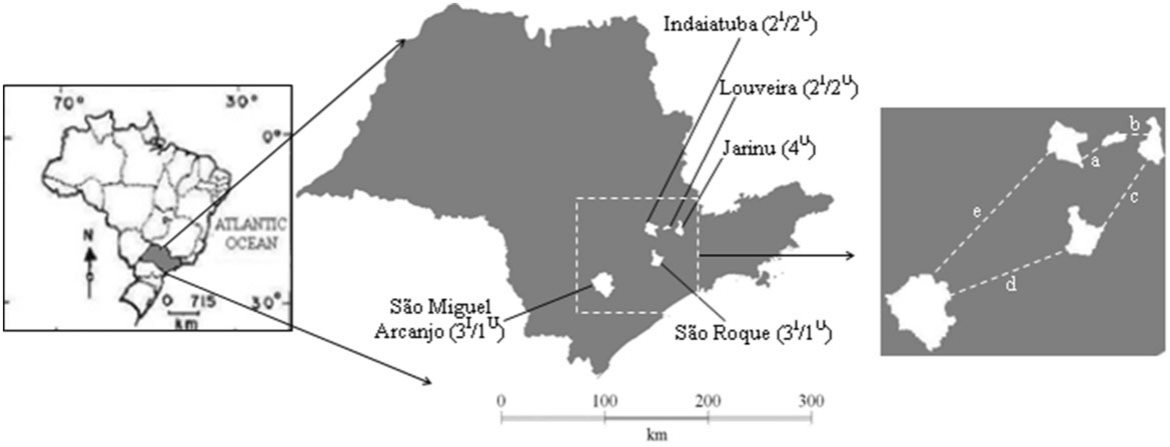
The location of the municipalities where collection took place in the Southeast Region of Brazil (São Paulo state) and an indication of the number of infested (I) or uninfested (U) vineyards (source: IBGE Instituto Brasileiro de Geografia e Estatística, 2011 modified). The letters a, b, c, d and e indicate the distance (km) between the municipalities sampled (a: 37.5; b: 33.0; c: 110.0; d: 117.0; e: 143.0).

The samples were collected in five municipalities that produce table grapes (Niágara Rosada) in southeastern Brazil (São Paulo state). These municipalities are located geographically close to each other: Indaiatuba (23°05′12″S, 47°13′06″W), Louveira (23°05′11″S; 46°57′02″W), Jarinu (23°07′22″S; 46°45′01″W), São Roque (23°31′45″S; 47°08′07″W), and São Miguel Arcanjo (23°52′42″S; 47°59′50″W; Fig. 1 ). The average temperature for the region during the collection period was 21°C, and the average rainfall was 128 mm ( Centro integrado de informações agrometeorológicas [Ciiagro] 2013 ). During the experimental phase, the cultivation routine was continued, i.e., insecticide application twice a year, weed removal using herbicides, and fertilizer application once a year.

Four vineyards in each municipality were selected, for a total of 20 study sites: 10 infested and 10 uninfested with the ground pearl ( Fig. 1 ). The collection sites within the same municipality were not more than 5 km apart. Infested vineyards were selected based on a thorough analysis of the grape vine roots of each plantation. In this analysis, the presence of nymphs, cysts, or female ground pearls was verified; the vineyard was considered to be infested when any stage of mealybug was recorded.

### Collection and Identification of Ants

**Fig. 2.**
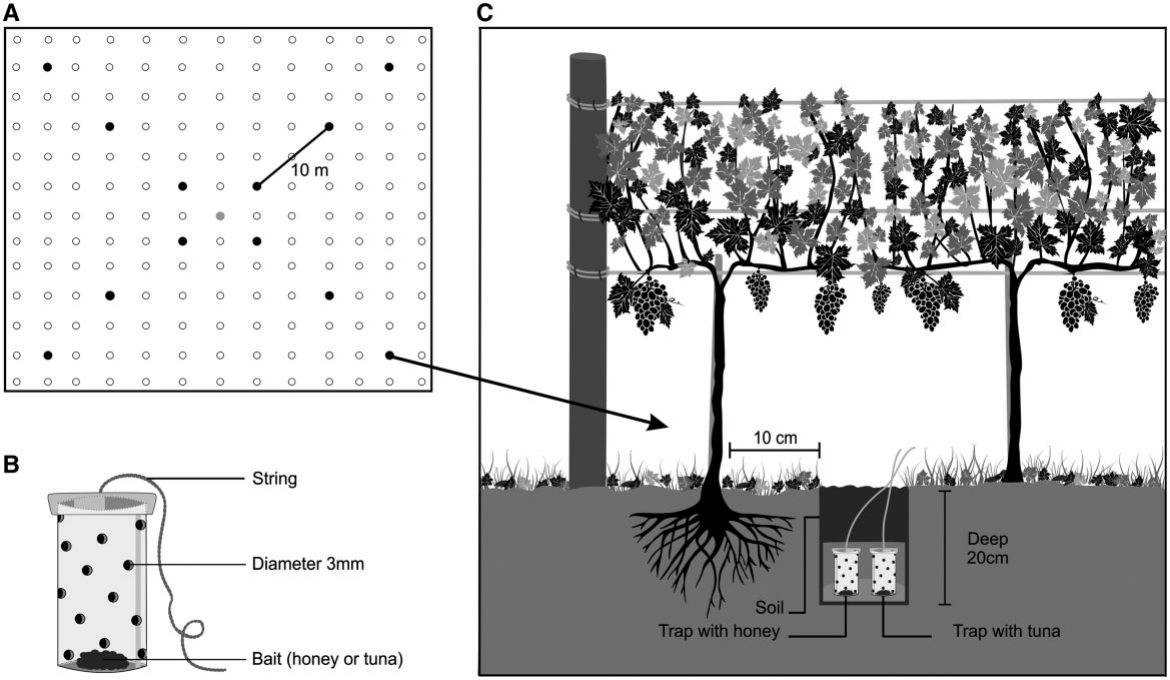
A representation of the distribution of subterranean traps in the vineyard (A); flask utilized as a trap (B); position of the subterranean trap in the ground (C). 
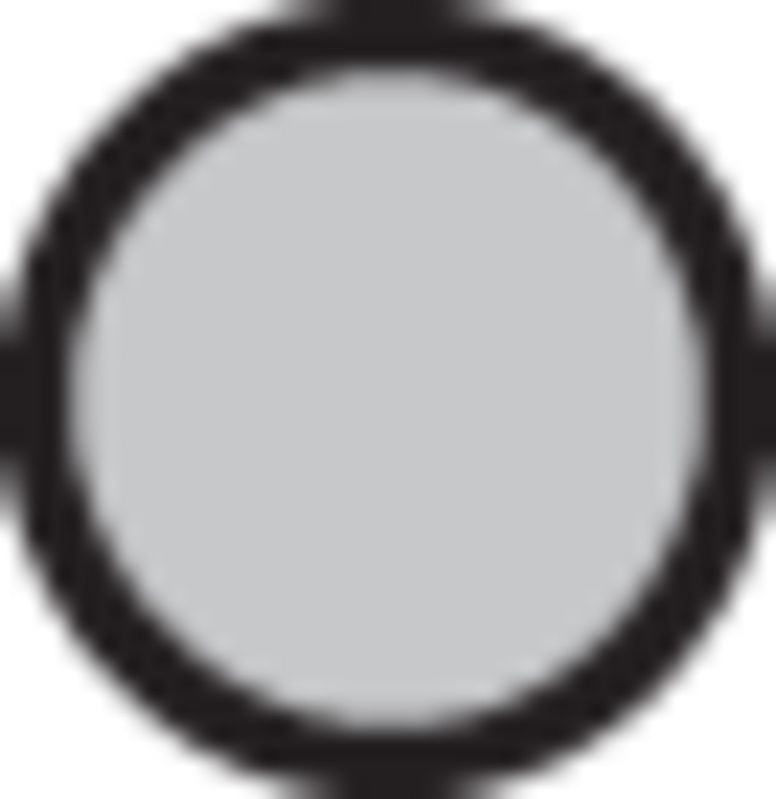
 , central cultivation area; 
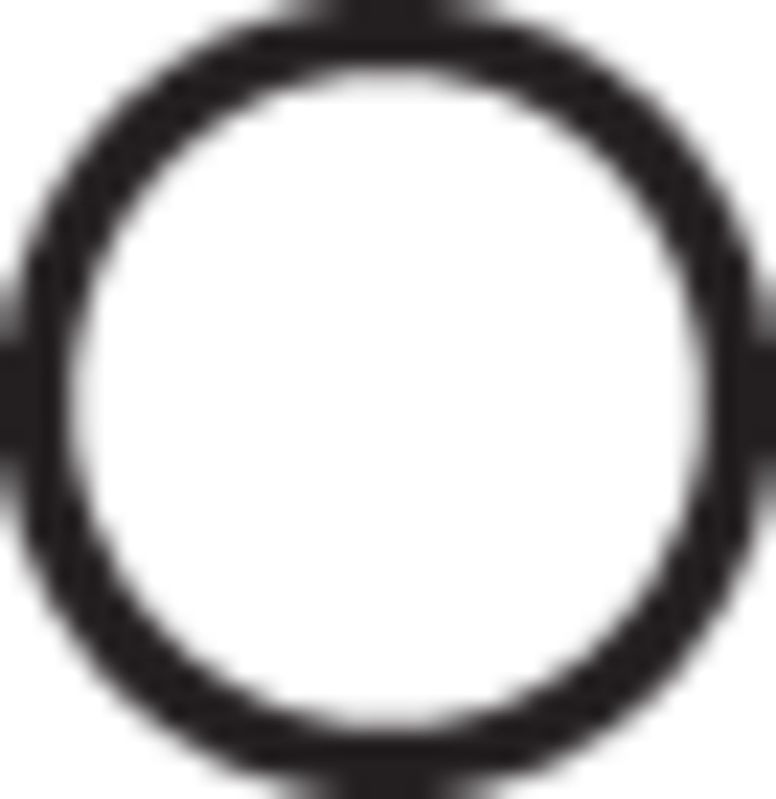
 , vine without a trap; 
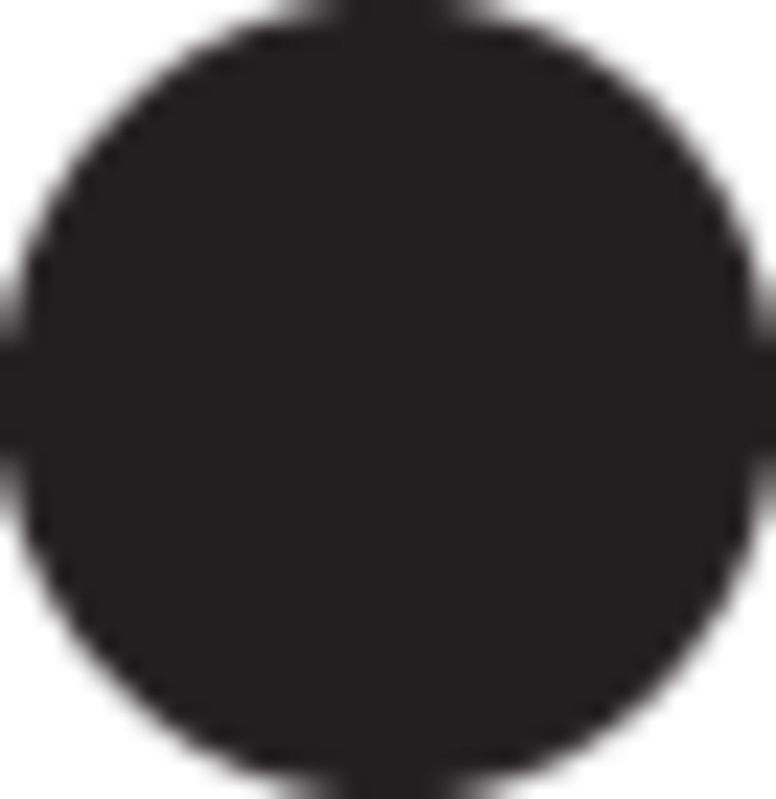
 , vine with a trap

In each vineyard, 12 holes were drilled to 20 inches deep with a manual excavator. The holes were equally spaced (=10 m; Fig. 2 A) and were dug close to the vine roots ( Fig. 2 C).

The ants were collected in the dry and rainy seasons and always in the morning using a trap made from a plastic flask (4.5 cm in height and 3.0 cm in diameter), perforated with 3-mm-diameter holes ( Morini et al. 2004 , Fig. 2 B and C).

Two traps were placed in each hole in the soil, one that contained crushed tuna mixed with oil in which the tuna was preserved (1 cm ^3^ ) and another that contained wild honey (1 cm ^3^ ; Fig. 2 C). Different types of attractants were used to capture a larger number of species of ants that forage beneath the soil surface. After 6 h in the ground, the traps were removed, and the material was separated according to collection point and bait. This procedure was followed for all the collecting expeditions.

The material was initially separated into subfamilies, then genera, and morphospecies ( Bolton 2003 , Bolton et al. 2006 ). For *Prenolepis* , the classification of Lapolla et al. (2010) was followed. Specimens for comparison were from the Alto Tietê Formicidae collection (Mogi das Cruzes University, UMC) and the Museum of Zoology of São Paulo University (Museu de Zoologia da Universidade de São Paulo). The codes for morphospecies followed those of the former collection. Voucher specimens were deposited at UMC (São Paulo).

The *Solenopsis* and *Linepithema* specimens were confirmed by molecular biology with the *cytochrome oxidase I* gene using the primers described by Ross et al. (1997) and Shoemaker et al. (2006) for the *Solenopsis* specimens and Hebert et al. (2004) for the *Linepithema* specimens.

### Data Analysis.

Comparisons among collection sites, infestation status, season, and bait type were performed using the number of species occurrences (presence and absence data) given that the object of interest was species richness and not the number of specimens. To analyze the diversity, the Shannon diversity indices ( *H* ′), equitability indices ( *E* ), and the Simpson index ( *D* ) were calculated using the BioDap software ( Thomas 2000 ).

The Mann–Whitney test was used to determine whether the observed richness was influenced by the season or the type of bait. Additionally, the Sorensen similarity index was applied to determine the similarity between the species in the infested and uninfested vineyards, between the dry and rainy seasons and between the different types of baits.

The association analysis was calculated for the five most common species sampled in the vineyards, using the Spearman’s correlation. BioEstat 5.0 software ( Ayres et al. 2007 ) was used for these tests, with a 5% level of significance.

## Results

**Fig. 3.**
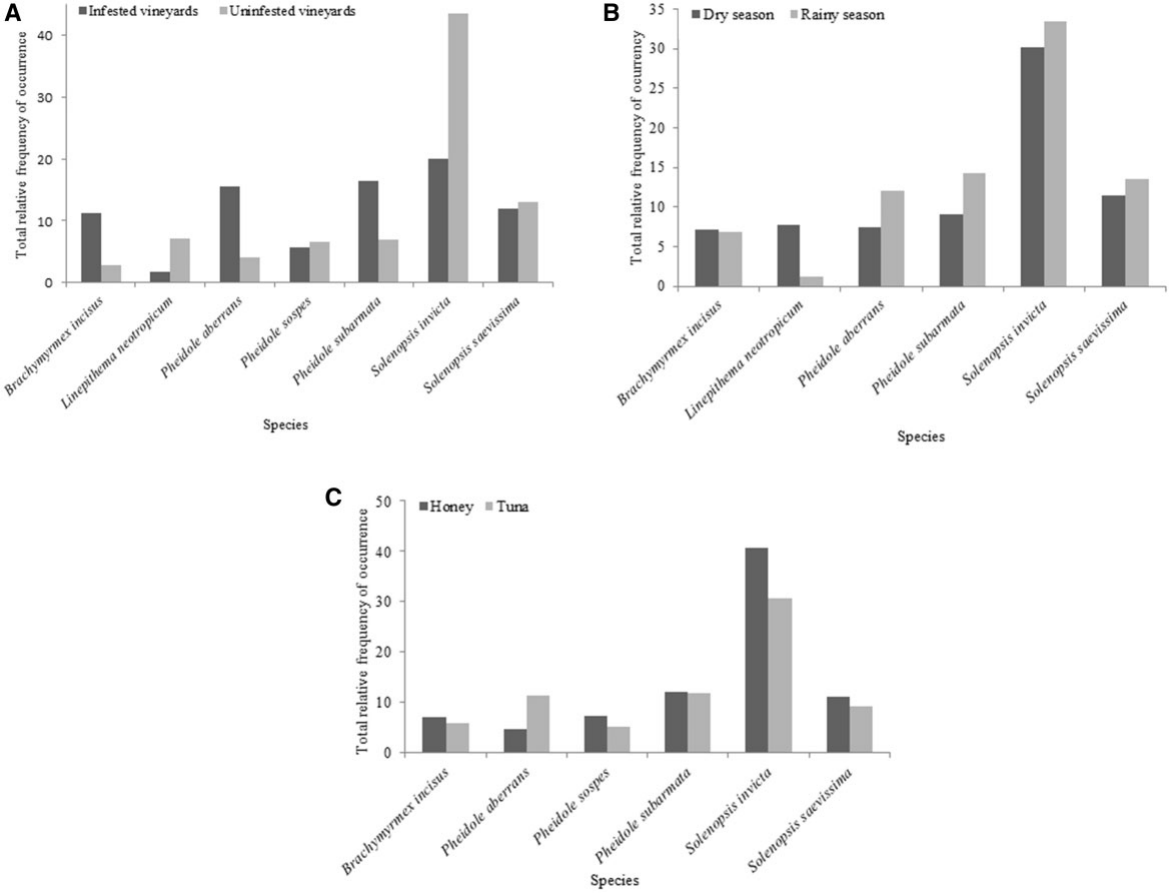
Most of the common species in the infested and uninfested vineyards (A) in the state of São Paulo during the dry and rainy seasons (B) and honey and tuna baits (C).

**Fig. 4.**
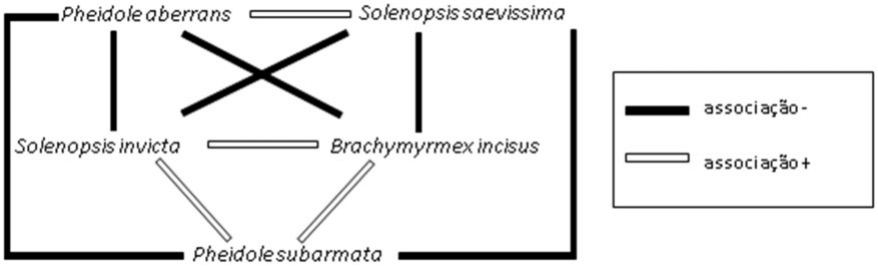
Structure of the ant community in vineyards in the state of São Paulo, based on the Spearman’s correlation.

In total, 86,748 ants were recorded, which were distributed among 6 subfamilies, 13 genera, and 20 species. Myrmicinae represented 53% of the species collected. *Pheidole* was the genus with the most richness, accounting for 31% of the species. The majority of recorded species belong to generalist taxa, except for *Labidus coecus* L *.* , which is cryptobiotic ( Table 1 ). *S. invicta* was the most common species in both the infested and uninfested vineyards ( Table 1 ; Fig. 3 A), irrespective of the season ( Fig. 3 B) and the type of bait ( Fig. 3 C). This species exhibited a negative association with *Solenopsis saevissima* (Smith) and *Pheidole aberrans* (Mayr) and a positive association with *Brachymyrmex incisus* F. and *P.subarmata* (Mayr; Fig. 4 ).

**Table 1.** The relative frequency of the occurrence (%) of the species recorded in vineyards infested or uninfested by *E. brasiliensis* in the Southeast Region of Brazil based on the season and the type of bait

Subfamily and species	Total relative frequency in vineyards	Infested vineyards					Uninfested vineyards		
		Dry	Rainy	Honey	Tuna	Dry	Rainy	Honey	Tuna
Dolichoderinae									
*Dorymyrmex* sp.1	0.5	2.4	—	1.3	0.5	—	—	—	—
*Linepithema neotropicum* Wild, 2007	5.2	1.2	2.3	1.8	2.2	14.3	—	6.7	5.2
*L. gallardoi* Brethes, 1914	0.5	—	0.6	0.1	0.2	0.5	1.0	1.5	0.5
Ecitoninae									
*Labidus coecus* Latreille, 1802	0.8	3.6	—	1.9	1.5	—	—	—	—
Ectatomminae									
*Ectatomma edentatum* Roger, 1863	2.6	2.4	5.2	3.8	3.9	1.3	2.1	1.7	1.9
Formicinae									
*Brachymyrmex incisus* Forel, 1912	6.4	11.4	11.0	11.5	9.2	3.0	2.6	2.6	2.6
*Camponotus melanoticus* (Emery)	1.3	1.8	—	0.6	0.5	2.1	1.0	2.6	1.5
*Nylanderia fulva* Mayr, 1862	5.2	5.4	2.9	2.5	4.4	3.8	8.8	0.4	5.7
Myrmicinae									
*Crematogaster* sp.1	0.5	1.2	0.6	0.6	1.0	0.4	—	—	0.8
*Pheidole aberrans* Mayr, 1868	9.1	12.6	18.5	8.3	19.4	2.5	5.7	0.9	3.4
*Pheidole sospes* Forel, 1908	6.2	6.6	4.6	7.0	4.4	8.4	4.6	7.3	6.0
*Pheidole* cf. *dione*	3.8	6.0	1.2	3.2	2.9	5.1	2.6	4.7	3.4
*Pheidole subarmata* Mayr, 1884	11.2	11.4	21.4	14.6	16.5	6.8	7.2	9.4	7.2
*Pheidole* sp.50	0.5	—	1.7	—	1.5	0.4	—	—	0.4
*Pheidole* sp.51	0.4	—	—	—	—	1.3	—	0.4	0.8
*Solenopsis invicta* Buren, 1972	32.8	23.4	16.8	32.5	22.3	37.1	50.0	48.9	39.2
*Solenopsis saevissima* Smith F., 1855	12.6	10.8	13.3	10.2	9.2	12.2	13.9	12.0	9.1
*Tetramorium* sp.1	0.1	—	—	—	—	0.4	—	0.4	—
Ponerinae									
*Odontomachus chelifer* Latreille, 1802	0.1	—	—	—	—	0.4	—	0.4	—
*Pachycondyla* sp.1	0.1	—	—	—	0.5	—	0.5	—	12.1
Shannon index ( *H* ′)		1.85					1.17		
Evenness ( *E* )		0.68					0.41		
Simpson index ( *D* )		0.2					0.47		

Infested and uninfested vineyards exhibited no significant difference in diversity indices ( *P* > 0.05; Table 1 ) nor in ant species richness. Seasonality also did not contribute to ant richness for either type of vineyard, and there was no preference based on the type of bait that was used ( Table 2 ).

**Table 2.** The total richness and the Mann–Whitney test for infested or uninfested vineyards based on the season and the type of bait

Richness	Vineyards		Mann–Whitney test
	Infested	Uninfested	
Total	16	18	*U* = 199.50; *P* = 0.49
Dry season	14	17	*U* = 191.00; *P* = 0.40
Rainy season	13	13	*U* = 188.50; *P* = 0.37
Honey	15	15	*U* = 180.00; *P* = 0.49
Tune	17	17	*U* = 171.00; *P* = 0.39

The ant communities were very similar in the dry and rainy seasons, independent of the presence of the ground pearl (Sorensen = 0.80) or whether honey or tuna baits were used (Sorensen = 0.88). The infested and uninfested vineyards were also similar in species composition of ants (Sorensen = 0.82).

## Discussion

This study is the first in Brazil to examine ant species in vineyards that are infested or uninfested by *E. brasiliensis* during different seasons. However, the data do not support the hypothesis that there is an association between mealybugs and the ant communities, let alone the ant species predominance. Unlike the state of Rio Grande do Sul, where *L. micans* is the dominant species and transports ground-pearl nymphs in infested vineyards ( Nondillo et al. 2010 ), in São Paulo, *S. invicta* is the prevalent species independent of the presence of mealybugs.

According to Pitts (2002) , there is no record of *S. invicta* occurring in the study region. However, Martins et al. (2012b) reported that their distribution is expanding in the Southeast Region of Brazil, which is consistent with the high frequency of this Solenopsidini ant genus in vineyards. Thus, even though this ant species can be associated with mealybugs ( Vinson and Scarborough 1991 ), its expansion into municipalities that grow grapes in the state of São Paulo cannot be related to the presence of *E. brasiliensis* . These results are reinforced by the similar frequency of *S. invicta* in the honey and tuna bait traps, i.e., their populations seem not to have increased due to the supply of the sugary food provided by mealybugs in vineyards.

Given its high invasive capacity, *S. invicta* is often negatively associated with other species in vineyards, especially *S. saevissima.* In another crop located in the eastern region of the state of São Paulo ( Oliveira et al. 2012 ), only *S. saevissima* was recorded. According to Martins et al. (2012b) , the expansion of *S. invicta* in southeastern Brazil is reaching the areas where *S. saevissima* is distributed; this may also be occurring in the region where this study was conducted.

In addition to *S. invicta* , the species *P. aberrans* , *P.subarmata,* and *B. incisus* were very common in infested vineyards, especially in the rainy season. As ground-pearl nymphs predominate during this season in the state of São Paulo ( Schmidt et al. 2009 ), and species of these genera of ants are associated with *E. brasiliensis* ( Soria et al. 1997 ), it is important to understand the biological associations between these taxa to control the spread of *E. brasiliensis.* Although honeydew release by ground pearls may be a factor in the ant dispersion of *E. brasiliensis* in the Southern Region of Brazil ( Nondillo et al. 2010 ), this is not the case in the vineyards of the southeast region. Other factors related to crop management should therefore be evaluated.

Thus, this study indicates that the ground pearl is not correlated with the composition of ant communities and the prevalence of certain species of ants in vineyards in the Southeast Region of Brazil. These results suggest a search for alternatives with respect to *E. brasiliensis* dispersion between plantations, including the use of machinery and grapevine seedlings that may be infested with mealybug cysts.
